# Metagenome-Assembled Genomes from a Microbiome Converting Xylose to Medium-Chain Carboxylic Acids

**DOI:** 10.1128/mra.01151-21

**Published:** 2022-03-28

**Authors:** Matthew J. Scarborough, Kevin S. Myers, Nathaniel W. Fortney, Abel T. Ingle, Timothy J. Donohue, Daniel R. Noguera

**Affiliations:** a Department of Civil and Environmental Engineering, University of Vermont, Burlington, Vermont, USA; b Great Lakes Bioenergy Research Center, University of Wisconsin-Madison, Madison, Wisconsin, USA; c Wisconsin Energy Institute, University of Wisconsin-Madison, Madison, Wisconsin, USA; d Department of Civil and Environmental Engineering, University of Wisconsin-Madison, Madison, Wisconsin, USA; e Department of Bacteriology, University of Wisconsin-Madison, Madison, Wisconsin, USA; Universidad Nacional Autónoma de México

## Abstract

There is growing interest in producing beneficial products from wastes using microbiomes. We previously performed multiomic analyses of a bioreactor microbiome that converted carbohydrate-rich lignocellulosic residues to medium-chain carboxylic acids. Here, we present draft metagenome-assembled genomes from this microbiome, obtained from reactors in which xylose was the primary carbon source.

## ANNOUNCEMENT

Using sludge from a well-characterized bioreactor producing hexanoic and octanoic acids from lignocellulosic residues (1–3), we sought to enrich for different community members by operating a new bioreactor with a synthetic medium that contained xylose as the primary carbon source at a concentration of 18,700 mg L^−1^. Biomass samples were collected after 96 (sample 16X), 102 (sample 17X), and 108 (sample 18X) days of bioreactor operation. DNA was extracted using a phenol-chloroform extraction method described previously (3). DNA aliquots of ∼3,000 ng for each of the three samples were shipped to the Joint Genome Institute (JGI, Berkeley, CA, USA; jgi.doe.gov) for sequencing.

The HiSeq 2500 system (Illumina, San Diego, CA) was used to generate 150-bp paired-end reads. Assembly was performed using JGI’s metagenome workflow (4) with jgi_mga_meta_rqc.py version 2.1.0. Trimmed, screened, and paired-end Illumina reads were corrected using BFC (5) version r181. Reads were assembled from single samples with SPAdes (6) version 3.11.1 with “—meta” mode enabled, and binning was performed using MetaBAT (7) version 0.32.5 with default settings to create metagenome-assembled genomes (MAGs). CheckM (8) version 1.0.11 was used with default settings to assess MAG quality, and GTDB-Tk (9) version 0.1.6 was used for taxonomic assignment using the GTDB (10) database release 202. GTDB-Tk was run using the following commands: gtdbtk identify --genome_dir ./--out_dir ./GTDBTk/--extension fasta --cpus 16; gtdbtk align --identify_dir ./GTDBTk/--out_dir ./GTDBTk/align --cpus 16. The software program dRep (11) version 3.2.2 was used to select representative MAGs from 31 MAGs obtained in this work plus 10 MAGs from prior analyses (1, 3). Default dRep settings were used except that the completeness threshold was set to 60%, the contamination weight was set to 0.5, and the *N*_50_ weight was set to 5. The phylogenetic tree was created using RAxML-NG version 0.9.0 with the following sequential commands: raxml-ng --parse --msa gtdbtk.bac120.user_msa.fasta --model LG+G8+F –prefix T1; raxml-ng --all --msa T1.raxml.rba --model LG+G8+F --prefix RAxML_ --threads 13 --seed 2.

The 16X assembly had an *N*_50_ value of 13.3 kb, an *L*_50_ value of 288, and an average coverage of 453× and produced 10 MAGs from 118,660,326 filtered reads. The 17X assembly had an *N*_50_ value of 5.6 kb, an *L*_50_ value of 722, and an average coverage of 402× and produced 9 MAGs from 111,508,430 filtered reads. The 18X assembly had an *N*_50_ value of 9.2 kb, an *L*_50_ value of 490, and an average coverage of 394× and produced 12 MAGs from 117,409,984 filtered reads. From the three assemblies, we obtained a total of 31 MAGs (completeness, >75%; contamination, <10%).

**FIG 1:**
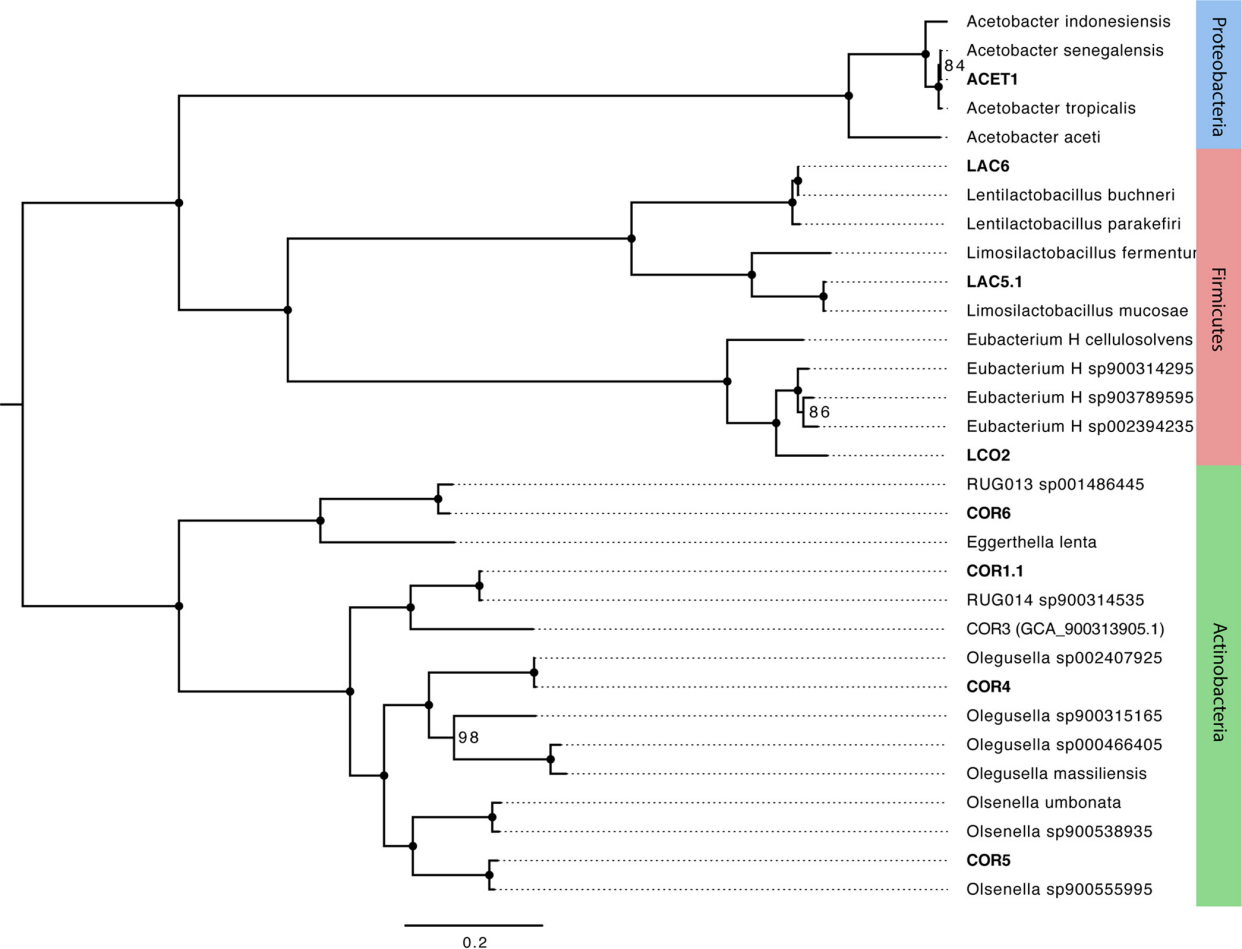
Phylogenetic tree constructed from recovered MAGs and related genomes. For each recovered MAG, three to five of the most closely related genomes were selected based on GTDB-Tk results (e.g., genus, species, average nucleotide identity [ANI], and alignment fraction [AF]). Genomes retrieved from NCBI were processed using GTDB-Tk to create concatenated sequences of 120 bacterial single-copy marker genes. The tree was constructed from the sequences using RAxML-NG (13) with 1,000 bootstraps. Filled circles indicate bootstrap values of 100, and all others are labeled as numbers. ACET, *Acetobacterales*; COR, *Coriobacteriales*; LAC, *Lactobacillales*; LCO2, *Lachnospirales*.

After dereplication, eight MAGs were selected as representative MAGs (Table 1), two of which are improvements to previously published MAGs (1, 3). COR1.1 is an update of COR1 (NCBI BioSample SAMN09651346), and LAC5.1 is an update of LAC5 (NCBI BioSample SAMN09651352). The other six MAGs represent new draft genome sequences of organisms within the GTDB (10) taxonomic orders *Acetobacterales* (ACET1), *Coriobacteriales* (COR4, COR5, and COR6), *Lactobacillales* (LAC6), and *Lachnospirales* (LCO2) (Fig. 1). Organisms related to *Acetobacterales*, *Coriobacteriales*, *Lactobacillales*, and *Lachnospirales* are regularly found via 16S rRNA gene amplicon sequencing (12) as abundant organisms during chain elongation. We provide the draft genome sequences of these organisms as a resource to further elucidate metabolic processes in this emerging biotechnological field.

**TABLE 1:** Summary of recovered metagenome-assembled genomes^*f*^

MAG ID	MAG name	Taxonomic classification (GTDB)^*a*^	Taxonomic classification (NCBI)	Comp. (%)^*b*^	Cont. (%)^*c*^	Size (Mb)	No. of scaffolds	Nearest GTDB^*a*^ genome (NCBI assembly no.)	ANI (%)^*d*^	AF (%)^*e*^	NCBI genome accession no.
ACET1	UW_Xyl_ACET1	s__Acetobacter senegalensis	*Acetobacter* sp.	99.5	0	3.1	36	GCF_001580995.1	97.56	89	JAJGAA000000000
COR1.1	UW_Xyl_COR1.1	s__UBA7748 sp900314535	*Atopobium* sp.	99.19	3.23	2.5	130	GCA_900314535.1	97.3	80	JAJGAB000000000
COR4	UW_Xyl_COR4	s__*Olegusella* sp002407925	*Olegusella* sp.	78.65	2.82	1.4	165	GCA_002407925.1	98.53	72	JAJGAC000000000
COR5	UW_Xyl_COR5	g__*Olsenella*	*Olsenella* sp.	100	6.45	2.7	46	GCF_009695875.1	92.91	84	JAJGAD000000000
COR6	UW_Xyl_COR6	g__RUG013	*Denitrobacterium* sp.	93.03	2.49	1.9	133	GCF_001486445.1	88.71	85	JAJGAE000000000
LAC5.1	UW_Xyl_LAC5.1	s__Limosilactobacillus mucosae	*Limosilactobacillus* sp.	99.18	0	2.0	17	GCF_001436025.1	96.49	88	JAJGAF000000000
LAC6	UW_Xyl_LAC6	s__Lentilactobacillus buchneri	*Lactobacillus* sp.	99.06	0	2.5	22	GCF_001434735.1	97.34	94	JAJGAG000000000
LCO2	UW_Xyl_LCO2	g__*Eubacterium*_H	*Eubacterium* sp.	77.14	2.12	2.5	384	NA	NA	NA	JAJGAH000000000

a GTDB, Genome Taxonomy Database (10); g_, genus name; s_, species name.

b Comp., completeness percentage estimated with CheckM (8).

c Cont., contamination percentage estimated with CheckM (8).

d ANI, average nucleotide identity with nearest GTDB genome estimated with GTDB-Tk (9).

e AF, alignment fraction with nearest GTDB genome estimated with GTDB-Tk (9).

f NA, not applicable. GTDB-Tk did not report a nearest genome because the AF with near genomes was below the default threshold of 65%.

### Data availability.

The raw metagenomic sequencing data are available under NCBI BioProject PRJNA518398 (sample 16X), BioProject PRJNA518399 (sample 17X), and BioProject PRJNA518400 (sample 18X). MAGs are available under NCBI BioProject PRJNA771338. Individual genome accession numbers for the MAGs are provided in Table 1.
